# Supplementation with *Spirulina platensis* Modulates Aortic Vascular Reactivity through Nitric Oxide and Antioxidant Activity

**DOI:** 10.1155/2019/7838149

**Published:** 2019-10-24

**Authors:** Aline de Freitas Brito, Alexandre Sérgio Silva, Alesandra Araújo de Souza, Paula Benvindo Ferreira, Iara Leão Luna de Souza, Layanne Cabral da Cunha Araujo, Gustavo da Silva Félix, Renata de Souza Sampaio, Maria da Conceição Correia Silva, Renata Leite Tavares, Reabias de Andrade Pereira, Manoel Miranda Neto, Bagnólia Araújo Silva

**Affiliations:** ^1^School of Physical Education, University of Pernambuco, Recife, Pernambuco, Brazil; ^2^Post-Graduation Program in Physical Education UPE/UFPB, Brazil; ^3^Physical Education Department, Health Sciences Center, Federal University of Paraiba, João Pessoa, Paraíba, Brazil; ^4^Laboratory of Studies of Physical Training Applied to the Performance and the Health, Health Sciences Center/Federal University of Paraiba, João Pessoa, Paraíba, Brazil; ^5^Federal University of Tocantins, Tocantinópolis, Tocantins, Brazil; ^6^Postgraduate Program in Natural and Synthetic Products Bioactive/Health Sciences Center, Federal University of Paraiba, João Pessoa, Paraíba, Brazil; ^7^Department of Biological Sciences and Health, State University of Roraima, Boa Vista, Roraima, Brazil; ^8^Department of Biophysics and Physiology, University of Sao Paulo, Institute of Biomedical Sciences, Sao Paulo, Sao Paulo, Brazil; ^9^Postgraduate Program in Nutrition Science/Health Sciences Center, Federal University of Paraiba, João Pessoa, Paraíba, Brazil; ^10^Pharmaceutical Sciences Department/Health Sciences Center, Federal University of Paraiba, João Pessoa, Paraíba, Brazil

## Abstract

The possible mechanism is involved in the effects of *Spirulina platensis* on vascular reactivity. Animals were divided into sedentary group (SG) and sedentary groups supplemented with *S. platensis* at doses of 50 (SG50), 150 (SG150), and 500 mg/kg (SG500). To evaluate reactivity, cumulative concentration-response curves were constructed for phenylephrine and acetylcholine. To evaluate the involvement of the nitric oxide (NO) pathway, aorta tissue was preincubated with L-NAME and a new curve was then obtained for phenylephrine. Biochemical analyses were performed to evaluate nitrite levels, lipid peroxidation, and antioxidant activity. To contractile reactivity, only SG500 (pD_2_ = 5.6 ± 0.04 vs. 6.1 ± 0.06, 6.2 ± 0.02, and 6.2 ± 0.04) showed reduction in phenylephrine contractile potency. L-NAME caused a higher contractile response to phenylephrine in SG150 and SG500. To relaxation, curves for SG150 (pD_2_ = 7.0 ± 0.08 vs. 6.4 ± 0.06) and SG500 (pD_2_ = 7.3 ± 0.02 vs. 6.4 ± 0.06) were shifted to the left, more so in SG500. Nitrite was increased in SG150 and SG500. Lipid peroxidation was reduced, and oxidation inhibition was increased in all supplemented groups, indicating enhanced antioxidant activity. Chronic supplementation with *S. platensis* (150/500 mg/kg) caused a decrease in contractile response and increase in relaxation and nitrite levels, indicating greater NO production, due to decreased oxidative stress and increased antioxidant activity.

## 1. Introduction

Nowadays, the practice of herbal medicine involves the use of more than 53,000 species of natural products for primary health care [1]. Several of these species are of aquatic origin and have drawn the attention of pharmaceutical research, considering that the aquatic environment is rich in natural resources and many biologically active compounds [2]. One group of aquatic organisms that has been highlighted due to its diverse biological activities is the blue-green algae. They belong to the phylum Cyanobacteria and family Spirulinaceae and are among the photosynthetic prokaryotes found in aquatic ecosystems. Certain species, including *Aphanizomenon flos-aquae*, *Spirulina platensis*, *S. maxima*, *S. fusiformis*, *Spirulina* sp, and *Nostoc comuna* var. sphaeroids Kutzing, have been consumed for centuries by humans [3]. Over time, these microalgae were used as traditional food by some Mexican, African, Native American, and Oriental people [3].

Clement [4] added that *S. platensis* and *S. maxima* have some advantages over other algae, such as pleasant taste, and do not present problems in your digestion and even apparent toxicity to humans, justifying the high consumption of *Spirulina* species by people. But populations of *S. platensis* are mostly found in waters with higher salt concentrations, such as a temporary nursery just before a drought, which makes this alga capable of adapting to different habitats and colonizing certain environments where other microorganisms would find it very difficult or even impossible to live [3], and such conditions characterize northeastern Brazil. *S. platensis* has a high reproduction rate, dividing three times a day, and in the same area, its cultivation can produce 125 times more protein than growing corn or 70 times more than raising cattle [5].

*S. platensis*, also known as *Arthrospira platensis*, is a blue-green alga with a helical shape and length of 0.2 to 0.5 mm. *S. platensis* has a high protein content (65 to 70% of its dry weight), contains all the essential amino acids, and represents a rich source of vitamin B_12_, minerals, essential fatty acids, and 15% complex carbohydrates [6, 7], plus photosynthetic pigments that display a variety of pharmacological properties [6, 8, 9].

Because of the above properties, there have been preclinical and clinical studies on the intake of *S. platensis*, showing that it can promote a decrease in serum triglycerides and low-density lipoproteins [10, 11], glycemic control [12, 13], reduction in allergic rhinitis [14, 15], growth of favorable intestinal microflora [16], anticancer [17], anti-inflammatory [13, 18, 19], antihypertensive [20, 21], and antioxidant [19, 21, 22] actions and reduction in endothelial dysfunction [20, 23, 24].

Among the effects attributed to *S. platensis*, antioxidant effects and regulation of endothelial function are important, considering that physiological changes characterized by an increase in vasoconstrictor response, decrease in vasodilator capacity, and increase in the production of reactive oxygen species due to decreased antioxidant enzyme activity are associated with cardiovascular risk factors such as hypertension [25–27].

Accordingly, various studies have investigated the pharmacological effects of a species of *Spirulina* and S*. maxima* and have demonstrated that it improves vascular tone [28, 29], while studies on *S. platensis* are still scarce. Huang et al. [30] reported the effect of supplementation with polysaccharides from *S. platensis* in diabetic rats and found that the contractile response was significantly reduced, while the relaxation response was increased in the aortic rings of these animals. Therefore, we tested the hypothesis that chronic dietary supplementation with freeze-dried powder of *Spirulin platensis* be able to improve the vascular reactivity, antioxidant activity, and endothelial function in the aorta of Wistar rats. Therefore, the objective of this research was to determine the effects of *S. platensis* on vascular reactivity in the isolated aorta of Wistar rats and the possible mechanisms involved in this response.

## 2. Materials and Methods

### 2.1. Drugs

Calcium chloride dihydrate (CaCl_2_·2H_2_O), potassium chloride (KCl), and sodium bicarbonate (NaHCO_3_) were purchased from Vetec (Rio de Janeiro, RJ, Brazil). Glucose (C_6_H_12_O_6_), magnesium sulfate heptahydrate (MgSO_4_·7H_2_O), hydrochloric acid (HCl), and monobasic potassium phosphate (KH_2_PO_4_) were from Nuclear (Porto Alegre, RS, Brazil). Sodium chloride (NaCl) was purchased from Dinâmica (Diadema, SP, Brazil) and acetylcholine chloride (ACh) from Merck (Brazil). Phenylephrine (PHE) was obtained from Pfizer (USA), and N*ω*-nitro-L-arginine methyl ester (L-NAME) was purchased from Sigma-Aldrich (Brazil). Ethylenediamine tetraacetic acid (EDTA) (1 : 250) came from BioTécnica-Advanced Biotechnology (Brazil), and carbogen mixture (95% O_2_ and 5% CO_2_) was obtained from White Martins (Brazil). All substances were weighed on an analytical balance, GEHAKA model AG 200 (Sao Paulo, SP, Brazil).

### 2.2. Animals

Wistar rats (*Rattus norvegicus*), weighing between 250 and 300 g, 2 months old, were obtained from the Professor Thomas George Bioterium from Universidade Federal da Paraíba (UFPB). The animals were maintained under controlled ventilation and temperature (21 ± 1°C) with water ad libitum in a 12 h light-dark cycle (light on from 6 to 18 h). Male Wistar rats (*Rattus norvegicus)* were used, weighing between 250 and 300 g; the animals were obtained from the Prof. Thomas George Bioterium of the Biotechnology Center of the Federal University of Paraiba (UFPB). All experiments were performed between 8 am and 8 pm, according to the guidelines for the ethical use of animals [31]. The experimental protocol was previously approved by Ethics Committee in Animal Use from CBiotec (CEUA/CBiotec) with certificate number 0511/13.

### 2.3. Preparation of and Supplementation with *Spirulina platensis*

*S. platensis* in powder form was obtained from Bio-engineering Dongtai Top Co., Ltd. (Nanjing, China) (lot no. 20130320). A sample was analyzed by the Pharma Nostra Quality Control Laboratory (Anapolis, GO) (lot no. 1308771A) to certify that the extract was obtained from *S. platensis* and was then prepared by Dilecta Manipulation Drugstore (Joao Pessoa, PB) (lot no. 20121025) for the preparation of the lyophilized powder.

The *S. platensis-*lyophilized powder was dissolved in saline solution (NaCl 0.9%) at doses of 50, 150, and 500 mg/kg. These values were based on the results of the experiments from other studies conducted with spirulina that investigated the anti-inflammatory, antioxidant, and relaxing effect [12, 22, 30, 32]. The supplements were administered for eight weeks for all doses (adapted, [19, 33]). Oral administration was daily between 12:00 pm and 14:00 pm, using stainless steel needles for gavage (BD-12, Insight, Ribeirão Preto, SP) and 5 ml syringes with an accuracy of 0.2 ml (BD, HIGILAB, Joao Pessoa, PB).

### 2.4. Groups and Supplementation

Animals were divided into sedentary saline (0.9% NaCl, control) and sedentary supplemented with *S. platensis* (50, 150, or 500 mg/kg) groups, using oral administration. Thus, the study was composed of the following groups with 20 rats randomly divided into 4 groups: sedentary saline group (SG, control) and sedentary groups supplemented with *S. platensis* at 50 mg/kg (SG50), 150 mg/kg (SG150), and 500 mg/kg (SG500). After eight weeks of intervention, the animals were anesthetized with thiopental sodium (100 mg/kg body weight) mixed with lidocaine (10 mg/ml) and then decapitated and after euthanized by cervical dislocation followed by exsanguination.

### 2.5. Aortic Ring Isolation

Animals were euthanized by guillotine, and the aorta was removed, cleaned of connective tissue and fat, immersed in physiological solution at room temperature, and bubbled with carbogen mixture (95% O_2_ and 5% CO_2_). In order to record the isometric contractions, aortic rings (3-5 mm) were individually suspended in organ baths (6 ml) by strap of stainless steel clips and isometric tension was evaluated by isometric force transducers (TIM-05 model), coupled to an AECAD04F model amplifier and connected to a digital acquisition system with AQCAD version 2.1.6 software to obtain the data and ANCAD software for analysis with a thermostatic pump model Polystat 12002 Cole-Parmer (Vernon Hills) that controlled the organ bath temperature.

The physiological solution of Krebs's solution was used and has the composition (in mM) as follows: NaCl 118.0, KCl 4.6, KH_2_PO_4_ 1.1, MgSO_4_ 5.7, CaCl_2_ 2.5, NaHCO_3_ 25.0, and glucose 11.0. The pH was adjusted to 7.4, and the ileum was stabilized for 1 h under a resting tension of 1 g at 37°C and bubbled with a carbogen mixture [34].

### 2.6. Concentration-Response Curves

The preparations were equilibrated for one hour, maintained under a rest tension of 1 g, and after the equilibration period, a contraction was induced with 3 × 10^−7^ M PHE, and during the tonic component, 10^−6^ M ACh was added to verify endothelium integrity [35]. The vascular endothelium was considered intact when the aortic rings showed relaxation equal to or greater than 50% [36]. In some aortic rings, the luminal surface was low rubbed with Krebs wet cotton to remove the endothelial layer. If the relaxation was equal to or less than 10%, the rings were considered devoid of functional endothelium.

To evaluate the contractile response of the rat aorta, after the verification of endothelium integrity, the preparations were washed, and after 30 min, cumulative concentration-response curves to PHE (10^−10^-10^−4^ M) in aortic rings were constructed for all groups, and to assess the relaxation response of the rat aorta, a new contraction with 3 × 10^−7^ M PHE was induced, during the tonic component of the contraction ACh (10^−11^–10^−4^ M) was added cumulatively to the aortic rings of all groups [33, 37].

To assess of the nitric oxide pathway, after the verification of endothelium integrity, preparations were washed, and after 30 min, the organ was preincubated with L-NAME (10^−4^ M), a competitive inhibitor of nitric oxide synthase (NOS) [33, 38, 39], and for 30 min, a cumulative concentration-response curve to PHE (10^−11^–10^−3^ M) was then induced in the aortic preparations of all groups.

The reactivity was evaluated based on the values of the maximum effect (*E*_max_) and the negative logarithm of the molar concentration of a substance that produced 50% of its maximal effect (pCE_50_) of both contractile agents, calculated from the concentration-response curves obtained.

### 2.7. Biochemical Measurements

After the animals were euthanized, 2 ml of blood was collected by cardiac puncture [40] and placed in test tubes containing anticoagulant (EDTA) to obtain plasma for determination of nitrite, MDA, and antioxidant activity [29, 40]. The samples were centrifuged at 1207 g for 15 min using a CENTRIBIO 80-2B-15ML centrifuge (Guarulhos, SP, Brazil). The plasma was transferred to Eppendorf tubes and stored at -20°C until analysis, and all analyses were performed within 7 days after blood collection. Nitrite, MDA, and antioxidant activity were measured in aorta fragments 8 mm long; these tissues were quickly removed, cleaned with Krebs solution to remove residual blood, placed in Eppendorf tubes, and stored in a freezer at -80°C until analysis.

### 2.8. Nitrite Assessment in Plasma and Aorta

Nitrite concentration was determined by the Griess method as described by Green [41]. Accordingly, the Griess reagent was prepared using equal parts of 5% phosphoric acid, 0.1% N-1-naphthyl ethylenediamine (NEED), and 1% sulfanilamide in 5% phosphoric acid and distilled water. A 500 *μ*l volume of plasma or tissue homogenate was added to 500 *μ*l of Griess reagent, and absorbance was read at 532 nm after 10 min. The blank used was 100 *μ*l of the reagent plus 100 *μ*l of 10% potassium phosphate buffer, and sodium nitrite (NaNO_2_) standards were made by twofold serial dilutions, to obtain 100, 50, 25, 12.5, 6.25, 3.12, and 1.56 mM solutions. Plasma and tissue samples were filtered prior to the assay. A Biospectro SP-220 spectrophotometer (Curitiba, PR, Brazil) was used for absorbance readings.

### 2.9. Assessment of Lipid Peroxidation

Lipid peroxidation was measured by the chromogenic product of 2-thiobarbituric acid (TBA) reaction with malondialdehyde (MDA) that is a product formed as a result of membrane lipid peroxidation [42]. Tissue samples were homogenized with 10% KCl in 1 : 1 proportions. Tissue homogenate and plasma samples (250 *μ*l) were incubated in a water bath at 37°C for 60 min. The samples were precipitated with 400 *μ*l of 35% perchloric acid and centrifuged at 26,295 g for 10 min at 4°C. The supernatant was transferred to new Eppendorf tubes, and 400 *μ*l of 0.6% thiobarbituric acid was added, followed by incubation at 95-100°C for 30 min. After cooling, the samples were read at 532 nm. Malondialdehyde concentration in plasma and tissue samples was determined using an MDA standard curve constructed using a standard solution (1 *μ*l of 1,1,3,3-tetramethoxypropane in 70 ml distilled water) diluted in a series of 250, 500, 750, 1000, 1250, 1500, 1750, 2000, 2250, 2500, 2750, and 3000 *μ*l of distilled water. In tissue, the absorbance values obtained were normalized to dry weight present in a given sample volume.

### 2.10. Evaluation of Antioxidant Activity

The procedure was based on the method described by Brand-Williams et al. [43], where 1.25 mg of DPPH (1,1-diphenyl-2-picrylhydrazyl radical) was dissolved in 100 ml of ethanol, kept under refrigeration, and protected from light (aluminum paper or amber glass). Then, 3.9 ml of DPPH solution was added with 100 *μ*l of the supernatant ileum homogenate on appropriate centrifuge tubes, vortexed, and allowed to stand for 30 min. They were centrifuged at 1207 g for 15 min at 20°C, and the absorbance of the supernatant was read at 515 nm. The results were expressed as percentage of the inhibition of oxidation, where AOA (antioxidant activity) = 100 − ((DPPH · R) T/(DPPH · R) B 100), where (DPPH · R) and (DPPH · R) B correspond to the concentration of DPPH^·^ remaining after 30 min, measured in the sample (T) and blank (B) prepared with distilled water. Tissue samples were homogenized with 10% KCl in 1 : 1 proportions. In tissue, the absorbance values obtained were normalized to dry weight present in a given sample volume.

### 2.11. Data Analysis

The functional results obtained were expressed as mean and standard error of the mean (S.E.M., *n* = 5), while the biochemical results were expressed as mean and standard deviation (S.D., *n* = 10). These results were statistically analyzed using two-way analysis of variance (ANOVA) followed by Bonferroni's posttest, and the differences between the means were considered significant when *p* < 0.05. pCE_50_ values were calculated by nonlinear regression [44], and *E*_max_ was obtained by averaging the maximum percentages of contraction or relaxation. All results were analyzed by the GraphPad Prism version 5.01 (GraphPad Software Inc., San Diego CA, USA).

## 3. Results

### 3.1. Effect of Supplementation with *S. platensis* on Contractile Response Induced by PHE in the Presence of Functional Endothelium in Isolated Rat Aorta

**Figure 1:**
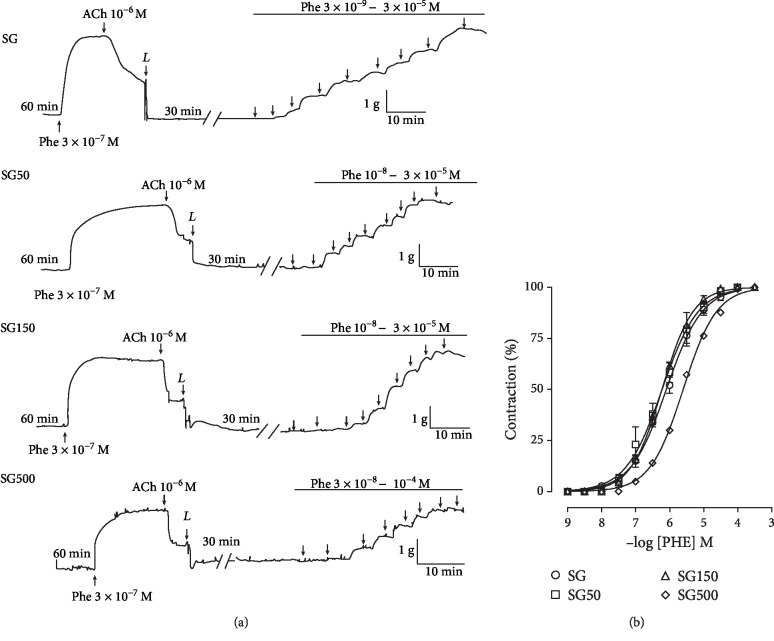
Representative traces (a) and contractile effect (b) of PHE in SG (○), SG50 (□), SG150 (∆), and SG500 (◊) groups in the rat aorta in the presence of endothelium. The symbols and vertical bars represent the mean and S.E.M., respectively (*n* = 05 indicates the number of samples per treatment). PHE: phenylephrine. Sedentary saline group (SG) and sedentary groups supplemented with *S. platensis* at 50 mg/kg (SG50), 150 mg/kg (SG150), and 500 mg/kg (SG500). Two-way ANOVA followed by Bonferroni's posttest, ^∗^*p* < 0.01 (SG vs. SG500), ^#^*p* < 0.01 (SG50 vs. SG500), ^†^*p* < 0.01 (SG150 vs. SG500).

Supplementation with *S. platensis* at doses of 50 (pCE_50_ = 6.2 ± 0.02) and 150 mg/kg (pCE_50_ = 6.2 ± 0.04) did not alter the contractile reactivity of the rat aorta to PHE compared with SG (pCE_50_ = 6.1 ± 0.06). However, rat treatment with 500 mg/kg *S. platensis* (pCE_50_ = 5.6 ± 0.04) shifted the cumulative concentration-response curve to PHE to the right, indicating a decrease in the contractile reactivity of the rat aorta with functional endothelium (Figure 1).

### 3.2. Effect of Supplementation with *S. platensis* on Contractile Response Induced by PHE in the Absence of Functional Endothelium in Isolated Rat Aorta

**Figure 2:**
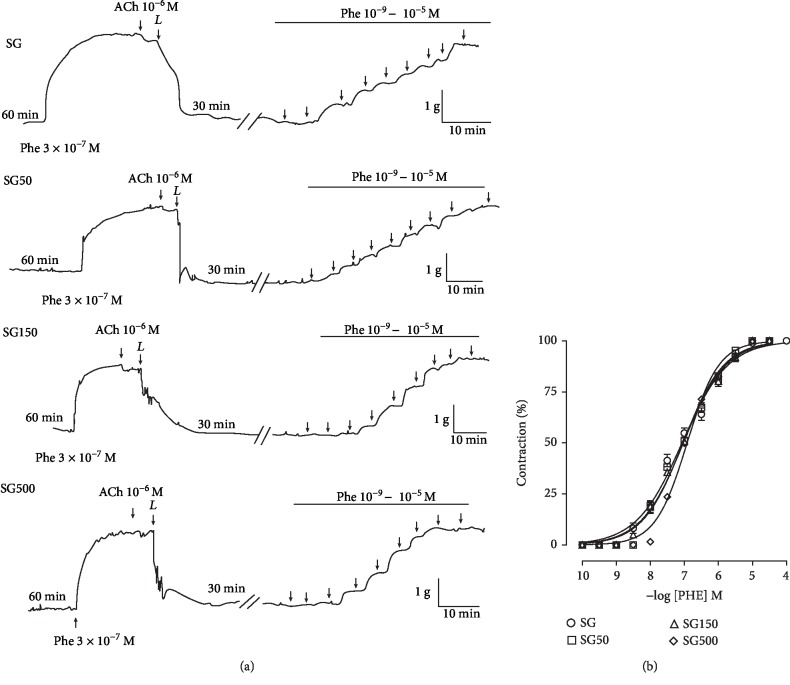
Representative traces (a) and contractile effect (b) of PHE in SG (○), SG50 (□), SG150 (∆), and SG500 (◊) groups in the rat aorta in the absence of endothelium. The symbols and vertical bars represent the mean and S.E.M., respectively (*n* = 05 indicates the number of samples per treatment). PHE: phenylephrine. Sedentary saline group (SG) and sedentary groups supplemented with *S. platensis* at 50 mg/kg (SG50), 150 mg/kg (SG150), and 500 mg/kg (SG500). Two-way ANOVA followed by Bonferroni's posttest.

Supplementation with *S. platensis* at doses of 50 (pCE_50_ = 7.0 ± 0.01), 150 (pCE_50_ = 7.1 ± 0.03), and 500 mg/kg (pCE_50_ = 7.0 ± 0.04) did not alter contractile reactivity to PHE in the rat aorta without functional endothelium compared to SG (pCE_50_ = 7.1 ± 0.04) (Figure 2).

### 3.3. Effect of Supplementation with *S. platensis* on Relaxation Induced by ACh in Isolated Rat Aorta

**Figure 3:**
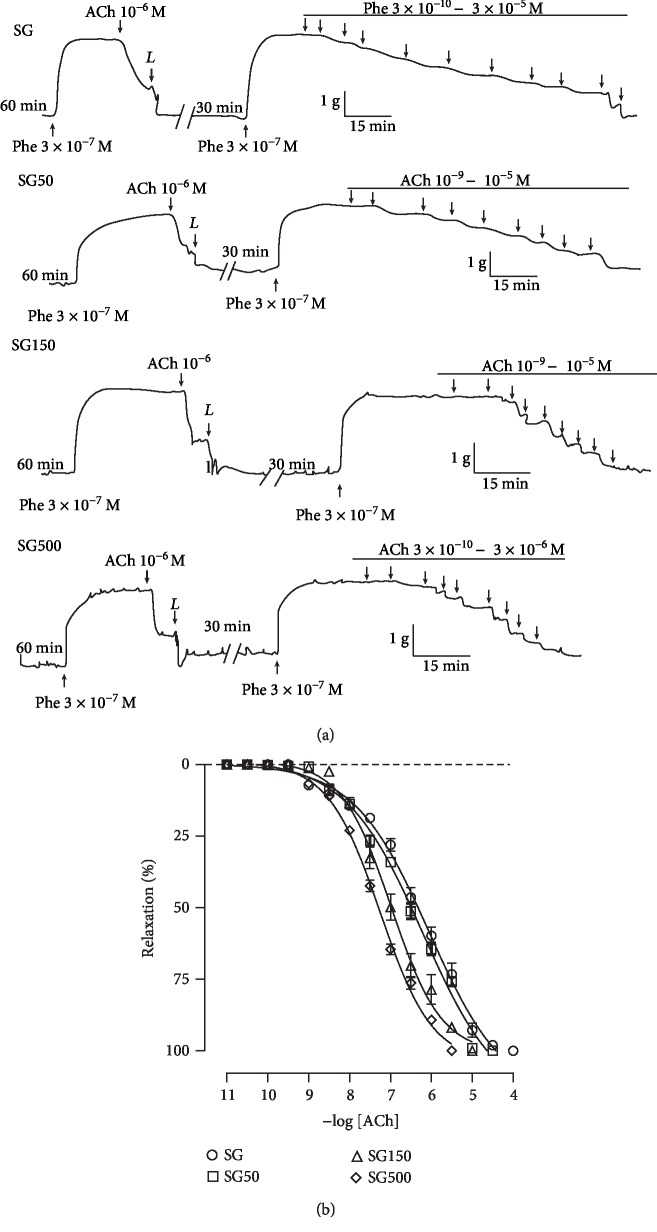
Representative traces (a) and relaxant effect (b) of ACh on the tonic contractions induced by 3 × 10^−7^ M PHE in SG (○), SG50 (□), SG150 (∆), and SG500 (◊) groups in the rat aorta in the presence of endothelium. The symbols and vertical bars represent the mean and S.E.M., respectively (*n* = 05 indicates the number of samples per treatment). Ach: acetylcholine. Sedentary saline group (SG) and sedentary groups supplemented with *S. platensis* at 50 mg/kg (SG50), 150 mg/kg (SG150), and 500 mg/kg (SG500). Two-way ANOVA followed by Bonferroni's posttest. ^∗^*p* < 0.01 (SG vs. SG150, SG vs. SG500), ^#^*p* < 0.01 (SG50 vs. SG150, SG50 vs. SG500), ^†^*p* < 0.01 (SG150 vs. SG500).

The relaxation curve induced by cumulative addition of ACh in the rat aorta with functional endothelium and precontracted with 3 × 10^−7^ M PHE from SG (pCE_50_ = 6.4 ± 0.06) was not altered in SG50 (pCE_50_ = 6.6 ± 0.1) (Figure 3). However, the relaxant potency of ACh was increased when the animals received supplementation with *S. platensis* at doses of 150 (pCE_50_ = 7.0 ± 0.08) and 500 mg/kg (pCE_50_ = 7.3 ± 0.02) (Figure 3), where a greater relaxant potency was found in the aorta of animals supplemented with *S. platensis* at 500 compared to 150 mg/kg. Supplementation with *S. platensis* did not alter the maximum effective relaxation of aortic rings (*E*_max_ = 100%).

### 3.4. Effect of Supplementation with *S. platensis* on Cumulative Contractions Induced by PHE in the Absence and Presence of L-NAME

**Figure 4:**
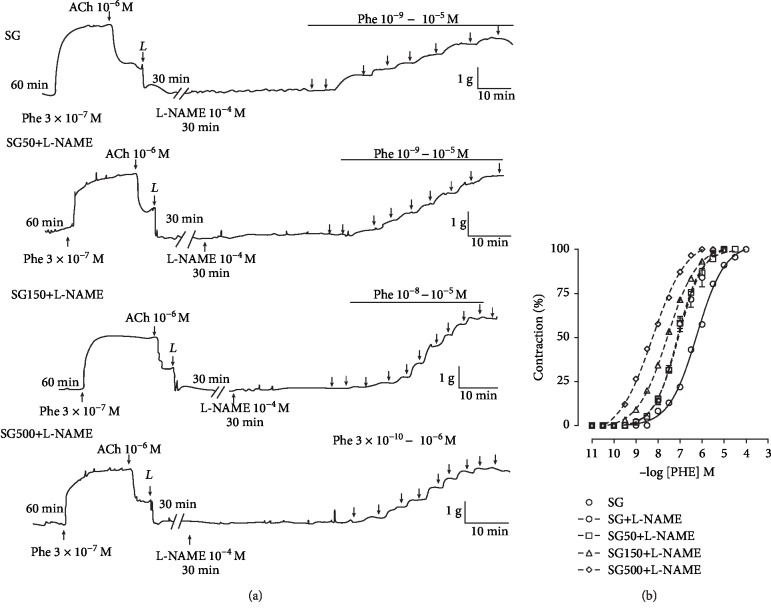
Representative traces (a) and contractile effect (b) of PHE in the presence of L-NAME in SG (○), SG50 (□), SG150 (∆), and SG500 (◊) groups in the rat aorta. The symbols and vertical bars represent the mean and S.E.M., respectively (*n* = 05 indicates the number of samples per treatment). L-NAME: N*ω*-nitro-L-arginine methyl ester. Sedentary saline group (SG) and sedentary groups supplemented with *S. platensis* at 50 mg/kg (SG50), 150 mg/kg (SG150), and 500 mg/kg (SG500). Two-way ANOVA followed by Bonferroni's posttest, ^∗^*p* < 0.001 (SG vs. SG+L-NAME, SG vs. SG50+L-NAME, SG vs. SG150+L-NAME, SG vs. SG500+L-NAME), ^#^*p* < 0.01 (SG+L-NAME vs. SG150+L-NAME, SG+L-NAME vs. SG500+L-NAME), ^†^*p* < 0.01 (SG50+L-NAME vs. SG150+L-NAME, SG50+L-NAME vs. SG500+L-NAME), ^§^*p* < 0.01 (SG150+L-NAME vs. SG500+L-NAME).

In the presence of L-NAME, cumulative concentration-response curves to PHE in SG (pCE_50_ = 7.1 ± 0.08) were shifted to the left in rats supplemented with *S. platensis* at doses of 50 (pCE_50_ = 7.1 ± 0.03), 150 (pCE_50_ = 7.6 ± 0.07), and 500 mg/kg (pCE_50_ = 8.2 ± 0.03) compared to SG in the absence of L-NAME (pCE_50_ = 6.1 ± 0.06) (Figure 4). In addition, treatment with 150 and 500 mg/kg *S. platensis* increased the contractile potency of PHE compared to SG and SG50 in the presence of L-NAME or SG in the absence of L-NAME (Figure 4).

### 3.5. Effect of Supplementation with *S. platensis* on the Production of Nitrite in Plasma and Rat Aorta

Plasma nitrite level was increased when the animals received supplementation with *S. platensis* at doses of 150 and 500 mg/kg, and the highest level was found in animals supplemented with 500 mg/kg compared to the lower doses (Table 1). The nitrite level in the aorta of animals that received supplementation with 150 mg/kg *S. platensis* increased compared to SG. However, the highest nitrite in the aorta was found in animals supplemented with 500 mg/kg (Table 1).

### 3.6. Effect of Supplementation with *S. platensis* on Lipid Peroxidation

Supplementation with *S. platensis* at doses of 150 and 500 mg/kg caused a significant reduction in lipid peroxidation in plasma. But the production of MDA in animals supplemented with 500 mg/kg *S. platensis* was lower than in those in SG, SG50, and SG150. Similarly, the production of MDA in the aorta was significantly reduced when the animals were supplemented with 150 and 500 mg/kg *S. platensis* compared to the lowest dose and control, but supplementation with the 500 mg/kg dose showed the greatest reduction in MDA production compared to the other groups (Table 2).

### 3.7. Effect of Supplementation with *S. platensis* on Antioxidant Activity

Only supplementation with *S. platensis* at the dose of 500 mg/kg increased the percentage of inhibition of the plasma oxidation when compared to SG, SG50, and SG150. Similarly, the percentage of inhibition of oxidation in the aorta was increased only when the animals were supplemented with the 500 mg/kg dose compared to other groups (Table 3).

## 4. Discussion

The present study demonstrated that chronic supplementation with *Spirulina platensis* at doses of 150 and 500 mg/kg caused an increase in the relaxation response to ACh and a decrease in the contractile reactivity to PHE in the rat aorta, and the mechanism of action seemed to involve the release of nitric oxide, reducing oxidative stress.

We found in the present study the supplementation with a lyophilized powder of *S. platensis*, which is one of the most common forms of this alga in numerous products sold in the market; *S. platensis* has the ability to modulate both in chronic vascular tone and lipid peroxidation in the aorta of Wistar rats.

Previous studies have shown both a reduction in contractile activity and enhanced relaxation in isolated aortic rings. However, most of these previous studies have investigated the *in vitro* effects of the ethanol extract of *S. maxima* in healthy and obese rats [28, 45]. In addition, Huang et al. [30] demonstrated that polysaccharides isolated from *S. platensis* alter vascular response in aortic rings from diabetic rats supplemented for six weeks. Carrizzo et al. [46] demonstrated which the peptidic hydrolyzate of *Spirulina platensis*, and in detail a decapeptide, is able to induce vasorelaxation both on normotensive mice and both on SHR through PI3K/Akt/eNOS-dependent mechanism.

Corroborating these results, our study also found that chronic dietary supplementation with *S. platensis* at doses of 150 and 500 mg/kg increased relaxation response to ACh, and that the 500 mg/kg dose also decreased contractile reactivity to PHE, which was totally dependent on the functional endothelium. The latter finding suggested that *S. platensis* altered the reactivity of the aorta promoting vascular relaxation in a dose-dependent manner that required the presence of factors derived from the vascular endothelium, even in healthy animals. In the study of Huang et al. [30], *S. platensis* polysaccharides were administered orally at doses of 12.261, 36.783, and 110.349 mg/kg in diabetic rats, and the authors reported that improvement in vascular reactivity occurred only with the highest dose. In our study, we also identified an increase in relaxation response and reduction in contractile response at a dose of 150 mg/kg, which was close to that active dose reported by Huang et al. [30], where the diabetic rats showed endothelial dysfunction. Our animals were healthy, and thus, the present results suggest that supplementation with the lyophilized powder of *S. platensis* is quite effective for the prevention of endothelial dysfunction.

In the present study, we also determined whether the reduction of contractile activity would be dependent on the mechanisms modulated by the endothelium. The results showed that the inhibition of contractile activity caused by *S. platensis* was endothelium-dependent, suggesting the participation of the NO pathway. The participation of endothelium in reducing contractile activity was accompanied by a significant increase in nitrite level in both plasma and aorta samples obtained from the animals supplemented with 500 mg/kg *S. platensis*; however, the dose of 150 mg/kg was still insufficient for this behavior to also reflect a reduction in vasoconstriction, confirming the dose-dependent effect. In addition, these data reinforced the importance of the endothelium in the effects observed with *S. platensis* and showed that these responses in smooth muscle were modulated by an increase in the bioavailability of NO. In view of these results, we can hypothesize that the constituents present in *S. platensis*, such as phycocyanin, can chronically increase the expression of endothelial nitric oxide synthase and consequently promote greater bioavailability of nitric oxide [47].

These results corroborated previous studies that found that *S. maxima* improved relaxant activity and reduced contractile response. Paredes-Carbajal et al. [28] showed that the ethanol extract of *S. maxima*, tested *in vitro*, caused a decrease in the contractile potency of PHE and an increase in relaxation response in rat aortic rings. In the endothelium-intact rings, these effects were blocked by L-NAME, suggesting that *S. maxima* extract increased the synthesis/release of NO [28]. Using the same pharmacological procedures, but in aortic rings of obese rats, Mascher et al. [45] and Juárez-Oropeza et al. [33] found similar results in relation to both relaxing and contractile response, such as the participation of the NO pathway [28, 33, 45]. Despite that possible mechanisms have been investigated in studies of *S. maxima*, in the study using the polysaccharides of *S. platensis*, no mechanism was investigated [30].

Oxidative stress was another mechanism that could also participate in the modulation of the response found in our results. The endothelial dysfunction is mainly a result of impaired NO availability, because a decrease in production and/or increase in degradation of NO leads to an increase in the production of reactive oxygen species such as O_2_^-·^, resulting in an increase in contractile response and/or reduced vasodilator response [48]. Accordingly, it was found that a significant decrease in malondialdehyde (MDA) in the plasma and aorta for the groups tested occurred with 150 and 500 mg/kg *S. platensis*. On the other hand, oxidation inhibition was also significantly increased in the plasma and aorta, but this increase occurred only at the dose of 500 mg/kg. Thus, these results reinforced the notion that reduced oxidative stress is essential for decreased contractile reactivity and increased relaxation response, as observed with *S. platensis* supplementation, suggesting that these responses are modulated by an increase in the antioxidant defense of the animals.

Previous data have shown that phycocyanin present in *S. platensis* stands out because of its high antioxidant capacity and scavenging of free radicals due to its stability [49] and inhibiting the formation of superoxide radicals by reducing the expression of the p22phox subunit of nicotinamide adenine dinucleotide phosphate oxidase [50, 51]. As carotenoids are essential for the regulation of superoxide dismutase and catalase and blocking free radicals by chelation of metal ions, they are able to prevent the lipid peroxidation [52]. Furthermore, B and E vitamins also act as antioxidants by capturing radicals and as metal-chelating agents [52].

However, it is important to observe that the techniques used to the determination of lipid peroxidation and antioxidant activity are accepted in the literature, but nonspecifically did not make it possible to understand which antioxidant compounds the *Spirulin platens* influenced.

In view of the changes in vascular reactivity and the benefits provided by dietary supplementation with *S. platensis*, it is worthwhile to investigate the potential of *S. platensis* as a nutraceutical food and dietary supplement to provide an alternative to the region's economy. However, further studies are necessary to extend our data, for example, in animals with certain diseases such as hypertension, diabetes, and/or obesity. Since the results of this study indicated that *S. platensis* could be used as a product with therapeutic targets, the next step proposed is to directly investigate the effect of dietary supplementation with *S. platensis* in humans to confirm that our findings are translatable or directly applicable.

## 5. Conclusion

This study evaluated the effect of feed supplementation with *S. platensis* on smooth muscle reactivity of the aorta isolated from healthy rats and the participation of the antioxidant system. It can be concluded that chronic supplementation with *S. platensis* at doses of 150 and 500 mg/kg alters the reactivity of the rat aorta resulting in a decrease in contractile response to PHE as well as an increase in relaxation response to Ach. The mechanisms underlying these effects on contractile and relaxation responses of the rat aorta involved presence of factors derived from vascular endothelium, increased bioavailability of NO, reduced lipid peroxidation, and increased antioxidant activity in both the plasma and aorta of healthy rats.

## Figures and Tables

**Table 1:** Concentration of nitrite in the blood plasma and aorta from the SG, SG50, SG150, and SG500 groups.

Groups	Nitrite (*μ*M)	
	Plasma	Aorta
SG	54 ± 11	28 ± 6
SG50	59 ± 8	31 ± 10
SG150	70 ± 7^∗^^#^	43 ± 6^∗^
SG500	88 ± 7^∗^^#†^	71 ± 7^∗^^#†^

The values represent the mean and S.D., respectively (*n* = 5 indicates the number of samples per treatment). Sedentary saline group (SG) and sedentary groups supplemented with *S. platensis* at 50 mg/kg (SG50), 150 mg/kg (SG150), and 500 mg/kg (SG500). Two-way ANOVA followed by Bonferroni's posttest, ^∗^*p* < 0.05 (SG vs. groups), ^#^*p* < 0.05 (SG50 vs. SG150 and SG500), ^†^*p* < 0.01 (SG150 vs. SG500).

**Table 2:** Lipid peroxidation in the blood plasma and aorta from the SG, SG50, SG150, and SG500 groups.

Groups	MDA	
	Plasma (nmol/l)	Aorta (*μ*M/g)
SG	8.4 ± 1.0	26 ± 7
SG50	8.2 ± 0.7	24 ± 2
SG150	6.8 ± 0.7^∗^	18 ± 2^∗^
SG500	5.0 ± 0.1^∗^^#†^	10 ± 3^∗^^#†^

The values represent the mean and S.D., respectively (*n* = 10 indicates the number of samples per treatment). Sedentary saline group (SG) and sedentary groups supplemented with *S. platensis* at 50 mg/kg (SG50), 150 mg/kg (SG150), and 500 mg/kg (SG500). Two-way ANOVA followed by Bonferroni's posttest, ^∗^*p* < 0.05 (SG vs. groups), ^#^*p* < 0.05 (SG50 vs. SG150 and SG500), ^†^*p* < 0.01 (SG150 vs. SG500).

**Table 3:** Percentage of oxidation inhibition in the blood plasma and aorta from the SG, SG50, SG150, and SG500 groups.

Groups	Oxidation inhibition (%)	
	Plasma	Aorta
SG	50 ± 5	12 ± 4
SG50	54 ± 4	14 ± 2
SG150	62 ± 3	17 ± 2
SG500	70 ± 2^∗^^#†^	27 ± 2^∗^^#†^

The values represent the mean and S.D., respectively (*n* = 10 indicates the number of samples per treatment). Sedentary saline group (SG) and sedentary groups supplemented with *S. platensis* at 50 mg/kg (SG50), 150 mg/kg (SG150), and 500 mg/kg (SG500). Two-way ANOVA followed by Bonferroni's posttest, ^∗^*p* < 0.05 (SG vs. groups), ^#^*p* < 0.05 (SG50 vs. SG150 and SG500), ^†^*p* < 0.01 (SG150 vs. SG500).

## Data Availability

The hypothesis and review data used to support the findings of this study are included within the article.

## Abbreviations

Ach: Acetylcholine
DPPH: 1,1-diphenyl-2-picrylhydrazyl radical
EDTA: Ethylenediamine tetraacetic acid
*E*_max_: Maximum effect
L-NAME: N*ω*-nitro-L-arginine methyl ester
MDA: Malondialdehyde
NO: Nitric oxide
NOS: Nitric oxide synthase
PHE: Phenylephrine
S.E.M.: Standard error of the mean
SG: Sedentary saline group
SG150: Sedentary groups supplemented with *S. platensis* at 150 mg/kg
SG50: Sedentary groups supplemented with *S. platensis* at 50 mg/kg
SG500: Sedentary groups supplemented with *S. platensis* at 500 mg/kg
TBARS: Thiobarbituric acid reactive substances.
